# One severe case of congenital toxoplasmosis in China with good response to azithromycin

**DOI:** 10.1186/s12879-021-06619-1

**Published:** 2021-09-06

**Authors:** Jiao Li, Jing Zhao, Xiaoyan Yang, Yang Wen, Liang Huang, Dan Ma, Jing Shi

**Affiliations:** 1 Department of Pediatrics, West China Second University Hospital, Sichuan University, Chengdu, 610041 Sichuan China; 2 Key Laboratory of Obstetric & Gynecologic and Pediatric Diseases and Birth Defects of Ministry of Education, Sichuan University, Chengdu, 610041 Sichuan China; 3 Department of Pharmacy/Evidence-based Pharmacy Center, West China Second University Hospital of Sichuan University, Chengdu, 610041 Sichuan China; 4 Department of Rehabilitation Medicine, West China Second University Hospital of Sichuan University, Chengdu, 610041 Sichuan China

**Keywords:** Congenital toxoplasmosis, Jaundice, Pancytopenia, Splenomegaly, Case report

## Abstract

**Background:**

Most infants infected with *Toxoplasma gondii* are completely asymptomatic at birth, yet they may develop ocular and neurological sequelae in the first few months of life. Cases of congenital toxoplasmosis with severe jaundice early after birth combined with pancytopenia and splenomegaly are extremely rare. Here, we report on a rare case of congenital toxoplasmosis presenting with severe jaundice and hemolysis early after birth combined with pancytopenia and splenomegaly.

**Case presentation:**

A male preterm infant with severe jaundice and splenomegaly was admitted to our department. Laboratory examinations revealed severe hyperbilirubinemia, increased reticulocytes, and pancytopenia. After comprehensive analysis and examination, the final diagnosis was congenital toxoplasmosis, and the infant was treated with azithromycin and subsequently trimethoprim-sulfamethoxazole. Regular follow-up revealed congenital toxoplasmosis in both eyes, which was surgically treated, while neurofunctional assessment results were unremarkable. In this case of congenital toxoplasmosis combined with severe jaundice, we treated the infant with two courses of azithromycin, followed by trimethoprim-sulfamethoxazole after the jaundice resolved. Clinical follow-up indicated that this treatment was effective with few side effects; thus, this report may serve as a valuable clinical reference.

**Conclusions:**

Timely diagnosis and adequate treatment are closely associated with congenital toxoplasmosis-related prognosis. Infants with congenital toxoplasmosis require long-term follow-up, focusing on nervous system development and ophthalmology.

## Background

Congenital toxoplasmosis is one of the most common types of intrauterine infection. The global incidence of congenital toxoplasmosis is approximately 190,100 cases annually, with an incidence rate of approximately 1.5 per 1000 live births [1]. The incidence of congenital toxoplasmosis is influenced by maternal infective status, climate, and socioeconomic conditions. There are evident geographic variations affecting infection rates according to the risk of acute toxoplasma infection in reproductive-age women. The global prevalence of latent *Toxoplasma gondii* infection (serum *T. gondii* immunoglobulin G (IgG) positive, immunoglobulin M (IgM) negative) in pregnant women was reported as 33.8% [2], while the prevalence of acute toxoplasma infection in pregnant women was 1.1%, with the highest prevalence in the Eastern Mediterranean region (2.5%) [3]. Higher incidence rates of congenital toxoplasmosis have been reported in some areas of South America [4, 5] (Brazil, 3.4 per 1000 live births [6]), the Middle East (Sahara area of Morocco, 8 per 1000 live births [7]), and low-income African countries (Benin, 3.4 per 1000 live births [1]). The prevalence of toxoplasma infection in China is low, presumably because most residents consume cooked food; however, it has increased over the past few decades, from 5.2% (1988–1992) to 7.9% (2001–2004) to 9.69% (2011–2017), which is attributed to socioeconomic development, an increase in international travel, and a rise in pet acquisition [8]. The prevalence of *T. gondii* is higher in southwest China owing to the consumption of raw or semi-raw meat. A national cross-sectional serosurvey conducted among Chinese women in the preconception period between 2010 and 2012 found that IgG and IgM seropositivity for *T. gondii* was 2.3% and 0.3%, respectively [9]. Although the average positivity rate for *T. gondii* IgG in pregnant women in China is approximately 2.4–5.0% [10], it can reach as high as 28.51% in certain areas [11]. The incidence of acute *T. gondii* infection diagnosed based on positive IgG and IgM enzyme-linked immunosorbent assay results exhibited a tendency to increase among Chinese pregnant women by approximately 0.3% in 2011 [12] and 1.5% in 2018 [3]. There is a current lack of epidemiological data on seroconversion rates during pregnancy since no systematic screening program for Chinese women of childbearing age exists. Similarly, national epidemiological data on congenital toxoplasmosis in China are scarce. The prevalence of congenital toxoplasmosis in China is estimated to be approximately 1.1 per 1000 live births based on the data from toxoplasma-specific IgM tests of pregnant women in China [1]. The seroprevalence of *T. gondii* in newborns is between 4.4 and 19.4% in some Chinese cities [13].

The most common manifestations of congenital toxoplasmosis are birth malformations or ocular and neurological sequelae shortly after birth. Early diagnosis and treatment of acute *T. gondii* infection during pregnancy can reduce the risk of vertical transmission and the disease severity of offspring. Additionally, timely intervention can effectively reduce the severe long-term sequelae in congenital toxoplasmosis infants [14]. The combination of sulfadiazine and pyrimethamine is recommended for the treatment of congenital toxoplasmosis [14]. However, sulfadiazine and pyrimethamine are not available in all regions, and the side effects of the drugs are intolerable in some cases. Additionally, reported cases with long-term follow-up results are rare. Herein, we described a rare case of congenital toxoplasmosis with the first symptoms being severe jaundice early after birth, combined with pancytopenia and splenomegaly. In addition, in this case, the infant was treated individually with azithromycin initially, followed by trimethoprim-sulfamethoxazole (TMP-SMX), and then comprehensively followed up.

## Case presentation

A male newborn with a gestational age of 33 weeks and birth weight of 2200 g was admitted to a local hospital because of preterm delivery and jaundice persisting for 1 day. He was delivered 1 day 14 h before admission to our department, with an Apgar score of 10 at 1, 5, and 10 min after birth. The newborn’s umbilical cord had been wrapped around his neck for 1 week at the time of delivery, and the amniotic fluid was clear without a history of intrauterine distress.

The mother did not receive regular antenatal care. However, fetal ultrasound during mid-pregnancy indicated an enhanced echo of the fetal bowel, peritoneal effusion, and separation of the left renal pelvis (0.6 cm). An amniotic fluid puncture test result was negative for cytomegalovirus DNA, and the gene chip (whole chromosome microarray analysis) revealed no abnormalities. The mother’s blood was type B, rhesus D (RhD) positive, and she experienced a 23-h premature rupture of her membranes, with no fever during delivery.

The newborn was admitted to a local hospital shortly after birth. The treatments administered included vitamin K1 and caffeine. The newborn presented with apnea approximately 4 h after birth and was treated with noninvasive ventilation. Approximately 8 h after birth, the newborn presented with jaundice; the transcutaneous concentration of bilirubin was 6.5 mg/dl. Approximately 12 h after birth, the level of bilirubin increased to 11.5 mg/dl. The newborn did not scream, exhibit convulsions or fever, or produce clay-colored stools and was administered phototherapy. Abdominal Doppler ultrasound indicated spleen enlargement. The total serum bilirubin concentration was 336.70 µmol/l. The newborn was subsequently transferred to our department because of persistent high bilirubin concentration despite intensive phototherapy. His 6-year-old sister had recovered from postnatal jaundice without undergoing phototherapy. The mother had eaten crayfish during the second trimester of the pregnancy. She did not raise or come into contact with animals and did not consume raw meat during the pregnancy. Further, she had no fever, rash, or enlarged lymph nodes during the pregnancy.

Findings of physical examination upon admission of the newborn to our department were as follows: temperature, 36.8 °C; heart rate, 112 beats/min; respiratory rate, 45 breaths/min; weight, 1910 g; head circumference, 29.5 cm; length, 45 cm; an immature appearance; poor responsiveness; severe yellow coloration of the skin of the whole body; petechiae and ecchymosis scattered over the body; and anterior fontanelle size, approximately 0.5 cm × 0.5 cm. The newborn’s abdomen was soft, and the lower margin of the liver was approximately 2 cm from the ribs. His spleen was enlarged, with the lower margin of the spleen approximately 6 cm from the ribs. The newborn exhibited a decreased muscle tone, and the primitive reflex was not observed. Neither of the testicles had completely descended into the scrotum. The remaining examination results were unremarkable.

Laboratory investigations were conducted. Chest radiography revealed an increased texture in both lungs. The white blood cell count was 6.7 × 10^9^/l (neutrophils, 42.8%; lymphocytes, 42.9%), the hemoglobin concentration was 122 g/l, the platelet count was 63 × 10^9^/l, and the C-reactive protein concentration was 0.7 mg/l. The reticulocyte percentage was 9.49%, and the absolute value was 0.2989 × 10^9^/l. The total serum bilirubin concentration was 405.8 µmol/l, and the indirect bilirubin concentration was 360.5 µmol/l. The newborn’s blood type was B, RhD positive. Glucose-6-phosphate dehydrogenase (G6PD) enzyme activity was normal, and the Coombs test result, coagulation test result, and blood and sputum cultures were negative. Hemoglobin electrophoresis results were normal. Cranial Doppler ultrasound revealed grade I intracranial hemorrhage. Cardiac Doppler ultrasound revealed acleistocardia, patent ductus arteriosus, and normal left ventricular systolic function.

The newborn received intensive phototherapy and exchange transfusion immediately after admission. The total serum bilirubin concentration decreased to 88.0 µmol/l, and the indirect bilirubin concentration was 23.5 µmol/l. However, the hemoglobin concentration and counts of white blood cells and platelets decreased; the lowest white blood cell count was 2.6 × 10^9^/l, the lowest neutrophil count was 0.41 × 10^9^/l, the lowest hemoglobin concentration was 81 g/l, and the lowest platelet count was 25 × 10^9^/l.

The combination of severe jaundice within 24 h after birth, progressive increase in total serum bilirubin concentration (mainly unconjugated bilirubin), low hemoglobin concentration, and high reticulocyte count led us to consider neonatal hemolytic disease. We excluded common etiologies, including blood group incompatibility and G6PD enzyme deficiency, following the hemolysis examination. As the infant presented with pancytopenia and splenomegaly, a congenital infection was considered. Therefore, we performed TORCH screening.

The first screen revealed a *T. gondii* IgM concentration of 14.0 AU/ml; an IgG concentration > 400 IU/ml; and positive results for herpes simplex virus IgG, cytomegalovirus IgG, and rubella virus IgG. Cerebrospinal fluid test results were negative. The second TORCH screen, 1 week later, revealed that the *T. gondii* IgM concentration had increased to 25.8 AU/ml.

**Fig. 1:**
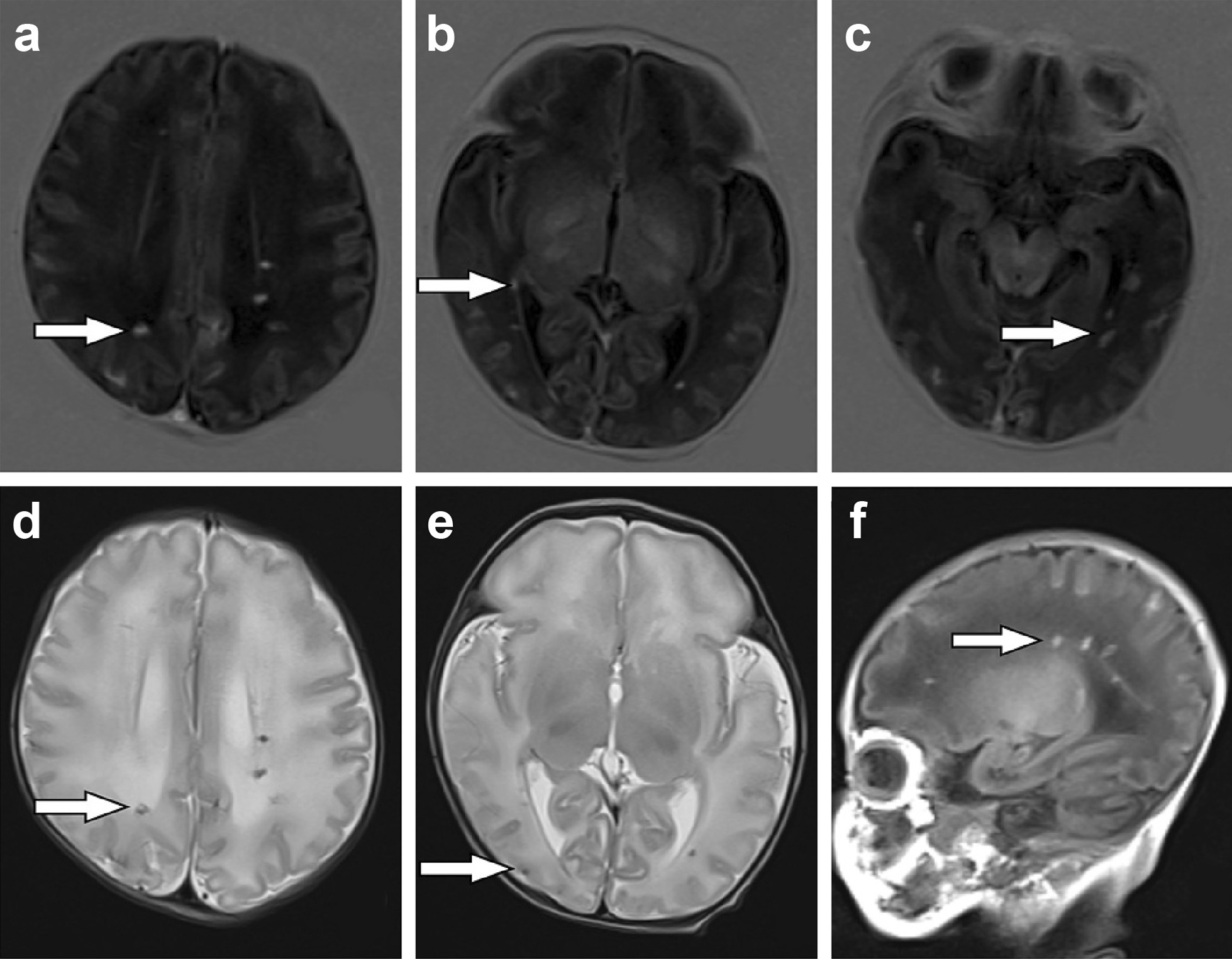
First cranial MRI of the infant

Since the parents refused computed tomography testing considering the risk of radiation toxicity, we performed magnetic resonance imaging (MRI) to evaluate the brain. The first cranial MRI (3 weeks after birth) revealed the following two main findings (Fig. 1): (1) There were abnormal signs of multiple nodules in the subcortical and deep white matter areas of both cerebral hemispheres, suggesting the possibility of an intrauterine infection (TORCH infection). (2) Both ventricles were widened. Both ears passed the automatic auditory brainstem response (AABR) test.

Punctate, small foci of abnormal signal intensity were scattered in the white matter areas of both cerebral hemispheres and in the subcortical white matter areas of both frontal, parietal, occipital, and right temporal lobes. The abnormal signals included a slightly increased signal intensity on T1-weighted images (T1WIs) (white arrows in panels a–c), a slightly lower signal intensity on T2-weighted images (T2WIs) (white arrows in panels d–e), and a slightly increased signal intensity on T1WIs (white arrows in panel f). Both ventricles were widened.

**Fig. 2:**
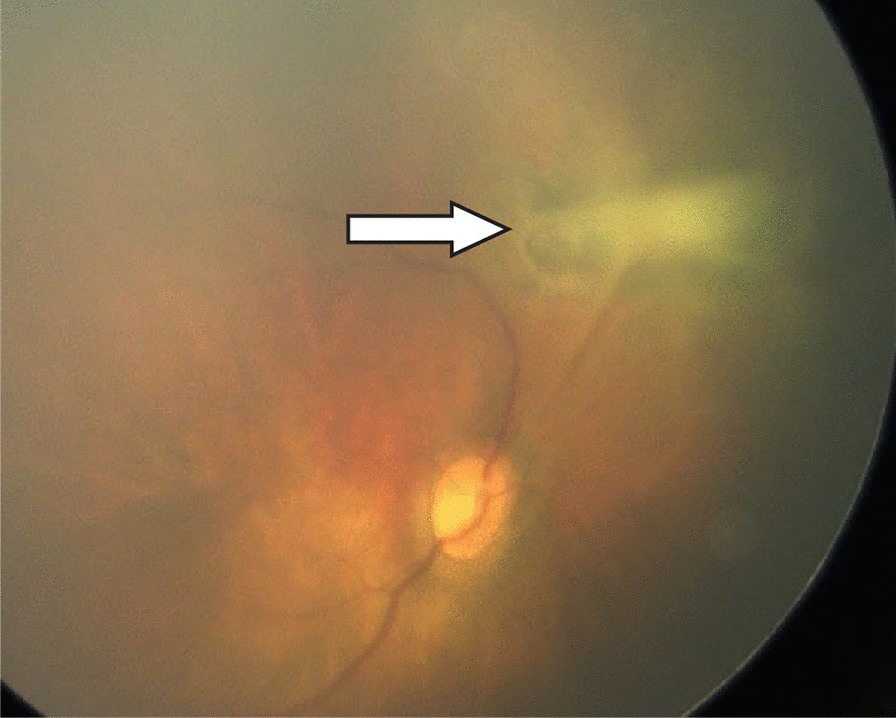
Neonatal screening by retinal examination of the infant’s right eye. White, space-occupying lesions were observed, exerting a slight tension on the retina above the optic disc (white arrow)

Neonatal retinal examination (Fig. 2) revealed bilateral stromal opacification, and that of the left eye was more pronounced. In the right eye, there was bleeding under the retina around the optic disc, as well as white, space-occupying lesions that exerted slight tension on the retina above the optic disc.

The first TORCH screen of the mother was conducted during the pre-pregnancy inspection, more than 5 months before the pregnancy, with positive results for IgG of herpes simplex virus, cytomegalovirus, and rubella virus, and negative results for *T. gondii* IgG and IgM. The second TORCH screen of the mother was conducted during the first trimester, yielding the same results. After delivery, the mother underwent a third TORCH screen, which revealed a *T. gondii* IgM concentration of 16.0 AU/ml and IgG concentration > 400 IU/ml. The affinity for *T. gondii* IgG was 0.32.

**Fig. 3:**
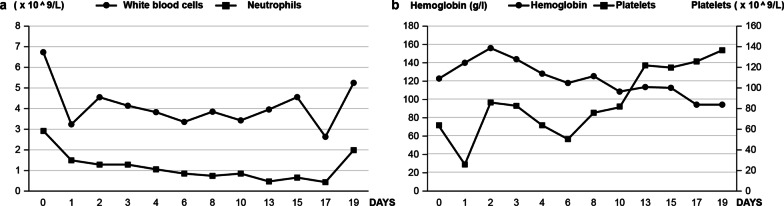
Routine blood examination of the infant during hospitalization. Left (**a**): white blood cell and neutrophil counts. Right (**b**): hemoglobin concentrations and platelet counts

Finally, the infant was diagnosed with congenital toxoplasmosis. Two courses of azithromycin were administered (day 1: 10 mg/kg, days 2–5: 5 mg/kg) after consultation with a clinical pharmacist and a pediatrician specializing in infectious diseases. The infant showed improvement and was discharged 23 days after hospitalization at a corrected gestational age of 37 weeks. Physical examination upon discharge revealed that the infant’s jaundice was clearly resolved, and the size of his spleen was obviously reduced. Laboratory investigations revealed a white blood cell count of 5.2 × 10^9^/l, neutrophil count of 1.94 × 10^9^/l, hemoglobin concentration of 93 g/l, and platelet count of 136 × 10^9^/l. Figure 3 illustrates the levels of white blood cells, neutrophils, hemoglobin, and platelets during hospitalization. Liver and kidney function tests were normal upon discharge.

Treatments after discharge included another course of azithromycin (day 1: 10 mg/kg, days 2–5: 5 mg/kg), followed by TMP-SMX (30 mg/kg/d), as suggested by a clinical pharmacist and pediatrician specializing in infectious diseases, from a corrected gestational age of 38 weeks to that of 11 months. The infant was regularly followed up. Both ears passed the AABR test 4 months after birth. During a follow-up ophthalmological examination, the infant was diagnosed with bilateral congenital toxoplasmosis, retinal detachment, accessory iris membranes, and complicated cataracts, as well as microphthalmos and vitreous hemorrhage of the left eye. Three ophthalmological operations were performed 3, 8, and 10 months after birth. Visual examination 11 months after birth revealed the following findings: right eye (spherical, − 2.00 D) and left eye (spherical, + 16.00 D; cylindrical, + 1.00 D; axial, 90°).

**Fig. 4:**
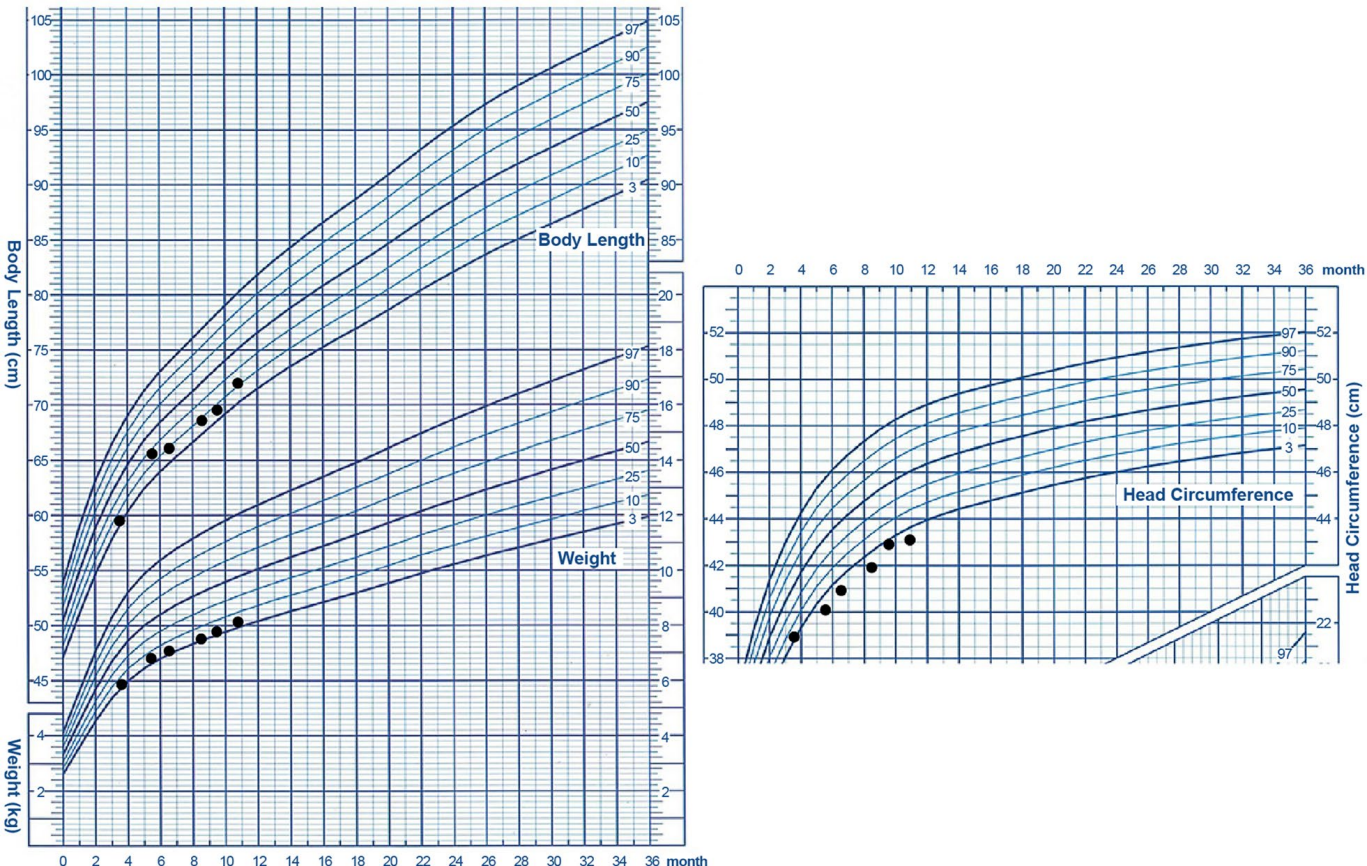
Physical development curve after discharge according to corrected age. Left: increase in weight and body length. Right: increase in head circumference

Figure 4 reveals the infant’s physical development curve after discharge, according to his corrected gestational age. His body weight fluctuated between the 3rd and 10th percentiles, body length fluctuated between the 10th and 25th percentiles, and head circumference fluctuated between the 0th and 3rd percentiles. The infant was regularly followed up with rehabilitation evaluation and guidance for rehabilitation provided to the family. Video electroencephalography 4 months after birth was normal. Table 1 presents the neurological evaluation of the infant.

**Table 1:** Neurological evaluation of the infant after discharge

Age (months)	Corrected age (months)	Evaluation test	Main conclusion
1+	41^+^1 weeks	TIMP	Motor developmental level was between 25 and 50%. Tracking ability was slightly impaired
4	3	BSID-2	Motor developmental level was normal. Cognitive developmental level was at 4.0%; sensitivity and range of tracking were slightly impaired. The current developmental level was slightly below normal
5	4	BSID-2	Motor developmental level was normal. Cognitive developmental level was at 14.4%. The current developmental level was slightly below normal
7	6	BSID-2	Motor developmental level was at 9.5%. Cognitive developmental level was at 15.9%. The current developmental level was slightly below normal
8	7	BSID-2	Motor and cognitive developmental level was normal
12	11	Griffiths	The level of gross movement, individual social capacity, listening, speaking, visual representation, and capacity in hand–eye coordination were all within the normal range

*TIMP* test of infant motor performance, *BSID-2* Bayley Scales of Infant Development-2

**Fig. 5:**
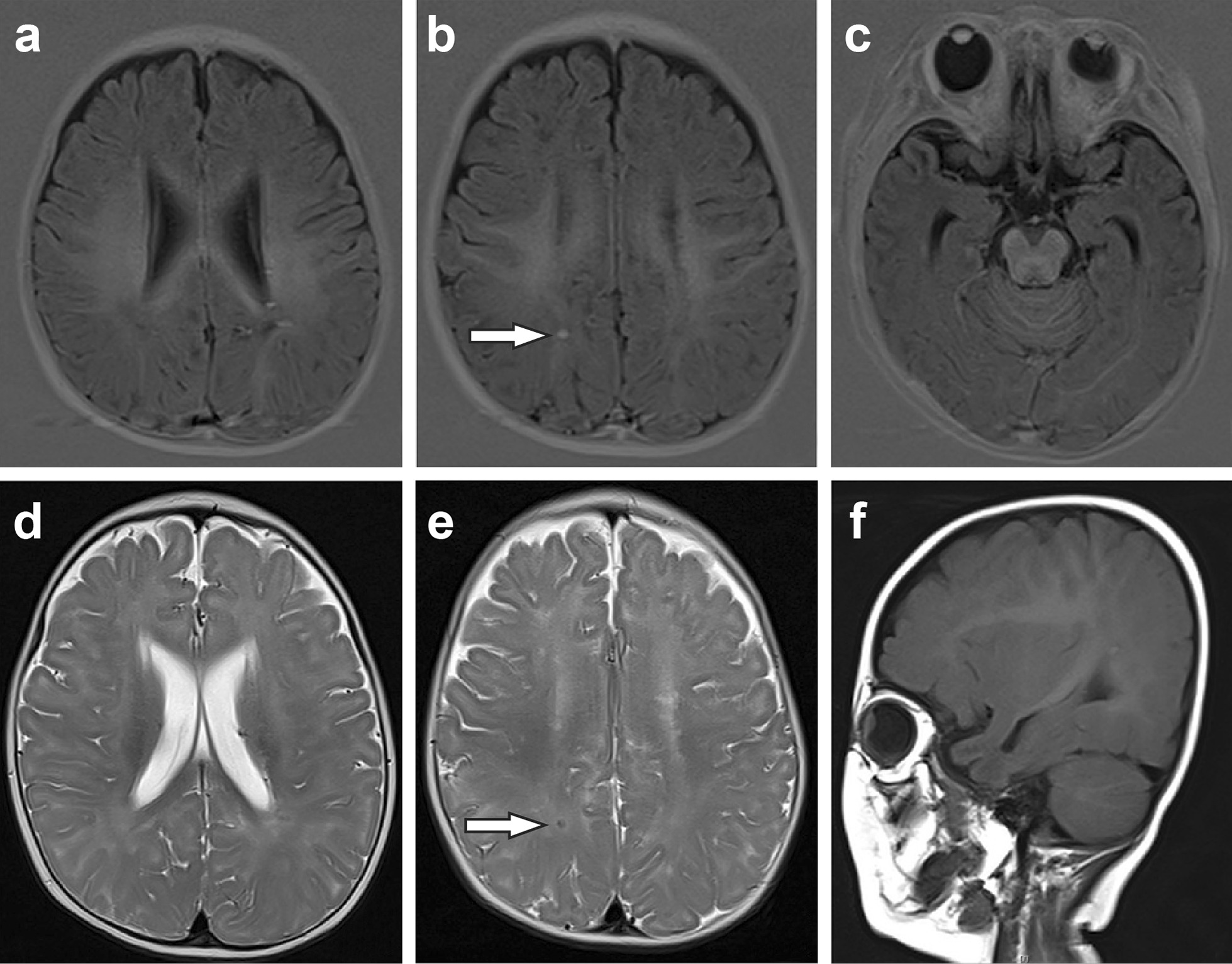
Second cranial MRI of the infant. A circular aberrant signal was observed in the white matter region of the posterior horn of the right lateral ventricle, with hyperintensity on T1-weighted images (T1WIs) (white arrows in panel **b**) and hypointensity on T2WIs (white arrows in panel **e**). There was no dilation of the ventricular system, no widening of sulci and fissures, and the midline structures were in the middle. Panels **a**–**c** refer to T1WIs, panels **d**, **e** refer to T2WIs, and panel **f** refers to T1WIs

The second cranial MRI, 6 months after birth, revealed one main finding (Fig. 5). There was a circular aberrant signal in the white matter region of the posterior horn of the right lateral ventricle, suggesting calcification (white arrow). Overall, the results from the second cranial MRI improved considerably from those of the first. In addition, routine blood test and liver function test results after discharge were normal. The infant underwent a serological test (including IgM and IgG of *T. gondii*) after completing a 1-year treatment according to a guideline from the USA [14]. The serological test result for *T. gondii* (both IgM and IgG) 13 months after birth (corrected age of 1 year) was negative.

## Discussion and conclusions

*Toxoplasma gondii* is an intracellular protozoan parasite. Congenital toxoplasmosis is caused by vertical transmission. In most cases, the pregnant mother is infected after contact with infected animals or intake of undercooked meat or food contaminated by oocysts; the infection reaches the fetus through the placenta [15]. Infection in the 3 months before pregnancy, reactivation of a latent infection in immunosuppressed women during pregnancy, and infection with a new strain of *T. gondii* in women who were previously infected can equally result in neonatal congenital toxoplasmosis. Only 30% of acutely infected women exhibit signs of the infection; these are nonspecific and include fever, fatigue, and cervical lymphadenectasis [16]. Diagnosis of toxoplasma infection during pregnancy depends mainly on serological screening or the detection of fetal ultrasonic abnormalities [17]. The maternal–fetal transmission rate is 30% for mothers with acute toxoplasma infection; it is 10–15% in the first trimester, 25% in the second trimester, and over 60% in the third trimester [18, 19]. The severity of fetal injury after toxoplasma infection depends on gestational age; the earlier the fetus is infected, the more severe the injury. Thus, although the maternal–fetal transmission rate in the early period of pregnancy is low, it may lead to serious consequences, including stillbirth, spontaneous abortion, and congenital malformation. In contrast, although the maternal–fetal transmission rate in late pregnancy is relatively high, the results are typically less severe [20]. The mother in this case did not have any definite contact with *Toxoplasma* during the pregnancy; TORCH screening findings in the first trimester were negative. She did not undergo a TORCH screen in the third trimester, and the prenatal follow-up did not reveal any unusual symptoms. A TORCH screen after delivery revealed substantial increase in *T. gondii* IgG and IgM, suggesting that the infant might have been infected by his mother in the second or third trimesters. In addition, the mother had consumed crayfish in the second trimester; if undercooked, that may have been the source of the primary infection. To date, guidelines for toxoplasma screening in pregnant women vary among countries. Since the prevalence of *T. gondii* infection in pregnant women is low in the USA and the UK (9.1% and 10%, respectively), and as there is a lack of standardized serological assays, routine screening of pregnant women is not recommended in those countries [21]. The guidelines of the American College of Obstetricians and Gynecologists recommend regular fetal ultrasound examinations. If ultrasonography indicates intrauterine infection (including ventriculomegaly, intracranial calcifications, microcephaly, and hepatosplenomegaly), a polymerase chain reaction test of the amniotic fluid for *Toxoplasma* testing is recommended. Amniocentesis should be performed after 18 weeks of gestation to reduce the rate of false negatives [18, 21, 22]. The prevalence of infection with *T. gondii* in pregnant women is higher in certain European countries, such as France (37%), Austria (33%), and Slovenia (34%); those countries have implemented mandatory prenatal screening for toxoplasmosis, every 1–2 months during pregnancy, to minimize transmission and injury to the fetus [21]. In China, TORCH screening is recommended for preconception examination; however, routine screening of pregnant women is not recommended. Targeted screening is recommended only for individuals who have special conditions, such as symptoms of infection or having been in close contact with infected individuals during pregnancy [23]. The mother of this case is a local resident of Chengdu city. Chengdu city is the capital of Sichuan Province in southwest China and is the fourth most populous city in China, with GDP ranking seventh among Chinese cities in 2020. In a survey of eight hospitals in China, the seroprevalence of *T. gondii* IgG/IgM of pregnant women in Chengdu was approximately 4.4%/0.4%, which is higher than that reported from Kunming (west China) 2.7%/0.34%, Yantai and Shanghai (east China) 0.68%/0.20%, Xiameng and Shenzhen (south China) 0.90%/0.22%, and Shenyang and Beijing (north China) 0.83%/0.31% [24]. Hot pot and crayfish are popular foods in Chengdu, and eating undercooked crayfish or meat in hot pots may explain the increased risk of toxoplasmosis infection among Chengdu women. According to a 1995 study of the Chengdu region, the seroprevalence of toxoplasma IgM in newborn infants was 1.07% [25]. However, more recent epidemiological data on the prevalence of congenital toxoplasmosis in the region are lacking. Given the lack of national epidemiological data on the incidence and prognosis of congenital toxoplasmosis in China, it is important to perform regular prenatal follow-up, especially fetal ultrasound examination, which plays a major role in the early discovery and diagnosis of fetal congenital toxoplasmosis.

Approximately 60–80% of infected infants are completely asymptomatic at birth but may gradually develop ocular and neurological symptoms in the first few months of life; the typical manifestation is a triad of retinochoroiditis, intracranial calcification, and hydrocephalus [14, 17]. The Systematic Review on Congenital Toxoplasmosis study group revealed that 19% of children had one or more clinical manifestations of *T. gondii* infection in the 1st year after birth and 14% and 9% had ocular and intracranial lesions, respectively [19]. Retinochoroiditis is the most common of these, with an incidence rate of 9–31% [17]. In this case, neonatal retinal examination revealed space-occupying lesions exerting a slight tension on the retina above the optic disc, indicating retinochoroiditis. Similarly, the infant was diagnosed with congenital toxoplasmosis in both eyes after being discharged.

The central nervous system is equally vulnerable to congenital toxoplasmosis, which may lead to meningitis, meningoencephalitis, cerebral parenchyma necrosis, diffuse calcification of brain tissue, and hydrocephalus. Patients may present with nervous system symptoms, such as vomiting, twitches, and lapsing into a coma, several months after birth. Severe cases may result in microcephaly and hydrocephalus after birth, as well as serious sequelae, such as cerebral palsy, worsening the prognosis [15, 26, 27]. The odds of infants experiencing brain lesions are closely related to the gestational age during which the mother had an acute infection. The estimated incidence of brain lesions was 30% if the mother was infected at 5 weeks of gestation, 10% at 20 weeks, and less than 5% at the third trimester [19, 28], and the severity of brain lesions decreased along with an increase in gestational weeks. In this case, no obvious nervous system symptoms were observed after birth; however, cranial MRI revealed a typical intrauterine change in infection. Neurological follow-up from birth to 12 months did not reveal any severe neurological sequelae. This indicated that the infant might have been infected with *T. gondii* during late pregnancy.

In addition, jaundice, hepatosplenomegaly, anemia, and thrombocytopenia are also classic features of congenital toxoplasmosis [26]. In this case, the infant had severe jaundice within 24 h after birth, accompanied with anemia, increased reticulocyte count, and splenomegaly. Congenital toxoplasmosis combined with hemolytic disease was considered because the other hemolysis tests were negative. Neonatal hemolytic disease is a rare complication of congenital toxoplasmosis. In an early report, neonatal death was ascribed to severe hemolytic anemia combined with congenital toxoplasmosis [29]. The jaundice of the infant in this case progressed after birth, which might have been related to hemolysis and toxoplasmosis hepatitis. It is highly recommended that, in the course of clinical diagnosis and treatment, congenital toxoplasmosis should be considered in the etiological analysis of early neonatal hemolytic disease, and relevant examinations should be performed. Thrombocytopenia is a common complication of congenital toxoplasmosis; neutropenia, however, has rarely been reported as a complication and may be related to immune destruction. After an extensive literature review, we did not find any reported cases of congenital toxoplasmosis with the first manifestations being severe jaundice and pancytopenia; thus, this report may serve as a valuable clinical reference.

Additionally, genotypes of *T. gondii* are the main factors affecting the clinical manifestations and outcomes of congenital toxoplasmosis. In Europe and North America, most of the patients are infected with types II and III of *T. gondii*, while more virulent atypical type of *T. gondii* are prevalent in South America [30]. Studies showed that the risks of ocular and intracranial lesions of congenital toxoplasmosis were much higher in South America than in Europe [4]. The dominating genotype of *T. gondii* in China is ToxoDB #9 (Chinese I or China I) and ToxoDB #10 (Type I), probably accounting for 90% of the strains of all samples (most from non-human) examined in China [31]. The relationship between these genotypes and the clinical manifestations and outcomes of congenital toxoplasmosis is unclear in China.

The diagnosis of congenital toxoplasmosis is mainly accomplished via serological tests because the isolation of *T. gondii* is difficult [26]. TORCH screening is the most commonly used assay for such a diagnosis, by detecting *T. gondii* IgG and IgM. We used the chemiluminescence method to measure anti-toxoplasma IgG and IgM levels during diagnosis and follow-up in this case. Since *T. gondii* IgG can pass through the placenta, it cannot be used to confirm the congenital nature of the infection. IgG transmitted from the mother to the infant gradually disappears within 6–12 months after birth; therefore, the concentration of IgG should be monitored dynamically in infants. Since IgM does not pass through the placenta because of its high macromolecular weight, a positive result indicates a congenital infection. In this case, the *T. gondii* IgM concentration increased in the neonate after birth, and his mother was equally positive for *T. gondii* IgM, supporting the diagnosis of congenital toxoplasmosis.

Timely treatment after birth can reduce the severity of the sequelae of congenital toxoplasmosis [32]. The American Academy of Pediatrics recommended pyrimethamine plus sulfadiazine plus folic acid for 12 months [14]. However, sulfonamides may cause serious adverse reactions, such as bilirubin encephalopathy and granulocytopenia, in the neonatal period. The infant in this case was born preterm with jaundice and granulocytopenia; thus, we did not immediately administer sulfonamides. Newer treatments, including azithromycin and atovaquone, are reportedly effective against *T. gondii* infection in its various stages [26]. Azithromycin has been reported as safe and effective in the pre- and postnatal treatment of *T. gondii* infection [33, 34]. Therefore, the infant in this case was treated with two courses of azithromycin following consultation with a pharmacist and pediatrician specializing in infectious diseases. TMP-SMX was used when the jaundice and granulocytopenia resolved, as pyrimethamine and sulfadiazine were unavailable in our area. A network meta-analysis revealed that TMP-SMX has potential as an alternative treatment for toxoplasmosis [35]. After discharge, routine blood, liver and kidney function, and auditory and neurological function test results were normal. Serologically following up on the IgG levels produced by *T. gondii* infections can help determine whether a favorable response to the treatment was obtained [36]. Thus, reduction in antibodies for *T. gondii* (both IgM and IgG) after completing the 1-year treatment indicates a good response to treatment.

Untreated congenital toxoplasmosis is related to a poor prognosis, and most survivors have severe neurological sequelae; timely and adequate treatment can improve the neurological and ocular prognosis. This case demonstrates the importance of early treatment. However, as neurological and ocular sequelae may still develop, long-term neurological and ophthalmological follow-ups are necessary.

## Data Availability

The datasets used and/or analyzed during the current study are available from the corresponding author on reasonable request.

## Abbreviations

AABR: Automatic auditory brainstem response
G6PD: Glucose-6-phosphate dehydrogenase
Ig: Immunoglobulin
MRI: Magnetic resonance imaging
RhD: Rhesus D
T1WI: T1-weighted image
T2WI: T2-weighted image
TMP-SMX: Trimethoprim-sulfamethoxazole
